# Correction: Evaluation of synergistic effect of entomopathogenic fungi *Beauveria bassiana* and *Lecanicillium lecacii* on the mosquito *Culex quinquefaciatus*

**DOI:** 10.1371/journal.pone.0323775

**Published:** 2025-05-12

**Authors:** Aditya Shankar Kataki, Francesco Baldini, Anjana Singha Naorem

The specie names “*Lecanicillium lecanii*” and “*Culex quinquefasciatus*” are misspelled in the article title. The correct title is: Evaluation of synergistic effect of entomopathogenic fungi *Beauveria bassiana* and *Lecanicillium lecanii* on the mosquito *Culex quinquefasciatus.* The correct citation is: Kataki AS, Baldini F, Naorem AS (2024) Evaluation of synergistic effect of entomopathogenic fungi *Beauveria bassiana* and *Lecanicillium lecanii* on the mosquito *Culex quinquefasciatus*. PLoS ONE 19(9): e0308707. https://doi.org/10.1371/journal.pone.0308707.

In Tables 2 and 3, there is a missing data. Please see the correct Tables 2 and 3 and their captions here.

**Table 2:** Larval mortality observed post 24 hours of treatment with *L. lecanii* fungal solution. Larval mortality upon treatment with different concentrations of combined fungal solution of *B. bassiana* and *L. lecanii.*

Sl. No.	Concentration of fungal solution (spores/ml)	Log concentration (spores/ml)	Mean Mortality ± S. D	C.M % (Abbott’s formulae)	Probit (P)
	Control (Distilled water)		8.34 ± 2.89	9	3.66
1	1 x 10^5^	5.00	88.34 ± 1.52	87.27	2.51
2	0.5 x 10^5^	4.69	84 ± 1	82.54	2.31
3	0.25 x 10^4^	3.39	62.34 ± 2.51	58.91	1.52
4	0.12 x 10^4^	3.07	54.34 ± 2.08	50.18	1.27
5	0.06 x 10^4^	2.78	42.34 ± 2.52	42.24	1.08
6	0.03 x 10^4^	2.47	32.34 ± 2.52	26.18	0.84

**Table 3:** Larval mortality observed post 24 hours of treatment with combined fungal solution of *B. bassiana* and *L. lecanii.*

Sl. No.	Concentration of fungal solution (spores/ml)	Log concentration (spores/ml)	Mean Mortality ± S. D	C.M % (Abbott’s formulae)	Probit (P)
	Control (distilled water)		8.34 ± 2.88	9	3.66
1	0.5 x 10^4^	3.69	80 ± 5	78.18	1.31
2	0.25 x 10^4^	3.39	78.34 ± 7.63	76.36	1.12
3	0.125 x10^4^	3.09	78.67 ± 6.50	76.72	0.91
4	0.06 x 10^3^	2.77	52.33 ± 2.51	47.99	0.66
5	0.03 x 10^3^	2.47	25 ± 5	18.17	0.38
6	0.015 x 10^3^	2.17	11.67 ± 2.88	3.63	0.10

The ORCID iDs is missing for the third author. Please see the author’s ORCID iD here:

- Author Anjana Singha Naorem’s ORCID iD is: 0000-0002-7937-689X

(https://orcid.org/0000-0002-7937-689X).
